# Using a Counting Process Method to Impute Censored Follow-Up Time Data

**DOI:** 10.3390/ijerph15040690

**Published:** 2018-04-05

**Authors:** Jimmy T. Efird, Charulata Jindal

**Affiliations:** Centre for Clinical Epidemiology and Biostatistics (CCEB), School of Medicine and Public Health, The University of Newcastle (UoN), Callaghan, NSW 2308, Australia; charulata.jindal@newcastle.edu.au

**Keywords:** counting process, censoring, Cox proportional-hazard regression, Kaplan-Meier, imputation, survival analysis

## Abstract

Censoring occurs when complete follow-up time information is unavailable for patients enrolled in a clinical study. The process is considered to be informative (non-ignorable) if the likelihood function for the model cannot be partitioned into a set of response parameters that are independent of the censoring parameters. In such cases, estimated survival time probabilities may be biased, prompting the need for special statistical methods to remedy the situation. The problem is especially salient when censoring occurs early in a study. In this paper, we describe a method to impute censored follow-up times using a counting process method.

## 1. Introduction

Ideally, censoring in a survival analysis should be non-informative and not related to any aspect of the study that could bias results [1,2,3,4,5,6,7]. For example, toxic side effects of an investigational drug may prompt the most ill patients to withdraw early from the study. Other patients may opt to leave before the intended end of a trial if the treatment is effective and they feel well. Even when censoring is non-informative (e.g., relocation to another city because of plant closure), by chance alone, it may still have a serious effect on estimated survival probabilities, especially if the dropouts occur early in the study.

In this paper, we present an example of early censoring to illustrate how the resulting survival probabilities may be biased. We then describe a method to impute censored follow-up times by rearranging the data as a counting process and generating jump-point plots. 

## 2. Materials and Methods

Imputing Censored Follow-Up Times

Let $\xi_{\left(j\right)}$ denote the follow-up time for the $j^{th}$ ordered observation, given a total of (n)  observations and (k) integer valued time (t) points. Accordingly,

(1) $$\left(t_{2}=t_{1}+1,\;t_{3}=t_{2}+1,\ldots ,\;t_{k}=t_{k-1}+1;t_{k}\geq n\right)$$

and

(2) $$t_{1}\leq \xi_{\left(1\right)}\leq \xi_{\left(2\right)}\ldots \leq \xi_{\left(n\right)}\leq t_{k.}$$

Then, for all values of $\xi_{\left(j\right)}$ and $t_{i}$ over their respective ranges (i.e., j=1 to n, i=1 to k), the counting process “indicator” variable (ζ) assumes the value 0 if $t_{i}\leq \xi_{\left(j\right)}$ and $\xi_{\left(j\right)}\;$ corresponds to a censored follow-up time (i.e., the outcome event, such as death, has yet to occurred by time $\xi_{\left(j\right)}$). Otherwise, when the observation denotes an event, (ζ) assumes the value 0 if $t_{i}<\xi_{\left(j\right)}$ and the value 1 if $t_{i}\geq \xi_{\left(j\right)}$ [8].

Next, we fit a multiple logistic regression model with (p+1)  covariates to the above counting process data, i.e.,

(3) $$P\left(\zeta =1|\beta_{0},\beta_{1},\beta_{2},\ldots \beta_{p+1}\right)=\frac{1}{1+e^{-\left\{\beta_{0}+\beta_{1}\left(t_{i}\right)+\beta_{2}\left(x_{1}\right)+\beta_{3}\left(x_{2}\right)+\cdot \cdot \cdot +\beta_{p+1}\left(x_{p}\right)\right\}}}$$

where the first covariate denotes time $\left(t_{i}\right)$ and the remaining (p) covariates $\left(x_{1,}x_{2,}\ldots ,x_{p}\right)$ represent outcome related risk factors. Model adequacy may be assessed by examining diagnostic plots, in lieu of standard goodness-of-fit tests, which would assume that the underlying counting process data are independently distributed [9,10,11,12]. In some cases, including higher-order or trigonometric terms into the logistic regression equation may improve model fit. 

A jump-point plot for a particular covariate pattern corresponding to a censored follow-up time $\left(\xi_{\left(j\right)}^{c}\right)$ may be obtained by plotting the model predicted values $\left(\hat{p}\right)$ against the time variable $\left(t_{i}\right)$. Let ${\tilde{\xi }}_{\left(j\right)}^{c}$ denote the value of $\left(t_{i}\right)$ that maps to $\hat{p}=0.50$ (i.e., maximum likelihood estimate of the mean jump-point observation). The imputed censored follow-up time is given as

(4) $${\tilde{\mathrm{imp}\xi }}_{\left(j\right)}^{c}=inf\left\{sup\left({\tilde{\xi }}_{\left(j\right)}^{c},\xi_{\left(j\right)}^{c}\right),\xi_{max}\right\},$$

where $\left(\xi_{max}\right)$ is the natural upper limit for a patient’s follow-up time. For example, in the case of cancer therapy, the maximum life expectancy of a patient rarely exceeds 101 years of age. For a patient aged 89 at diagnosis, we see that $\xi_{max}$ is computed as (101 minus 89). The original censored follow-up times are then replaced with ${\tilde{\mathrm{imp}\xi }}_{\left(j\right)}^{c}$. However, it is important to note that these follow-up times are still treated as censored values rather than events when computing survival probability estimates.

Assuming Martingale independence and considering the event times to be binomially distributed (i.e., the probability $\left(\pi_{\nu }\right)$ of an event $\left(\varsigma_{\nu }\right)$ occurring in a particular risk set (ν) is equal to its expectation $E\left(\varsigma_{\nu }\right)$ divided by the risk set size $n_{\nu }$ (accounting for censored and event times), with corresponding variance equal to $n_{\nu }\pi_{\nu }\left(1-\pi_{\nu }\right)$), it follows that the resulting data be may analyzed using standard methods for handling censored time-to-event observations (e.g., Kaplan-Meier and Cox proportional-hazards models) [13,14]. Here, the sampling variability for estimating the imputed censored follow-up times is absorbed into the sampling variability for each binomially distributed event time, given that the estimates are asymptotically consistent and adhere to certain regularity conditions.

## 3. Results

### 3.1. Kaplan-Meier (Product-Limit) Example

**Figure 1:**
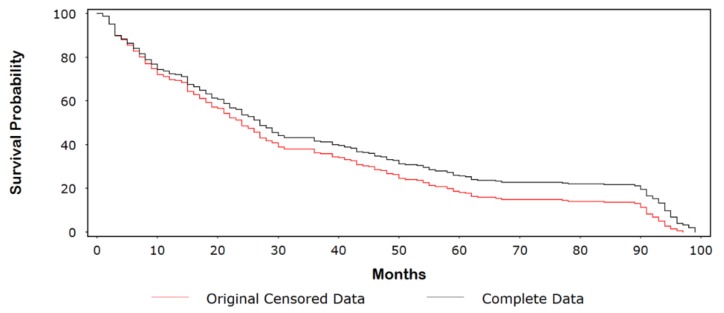
Kaplan-Meir (KM) plot comparing the original censored data with the dataset of complete follow-up times.

**Figure 2:**
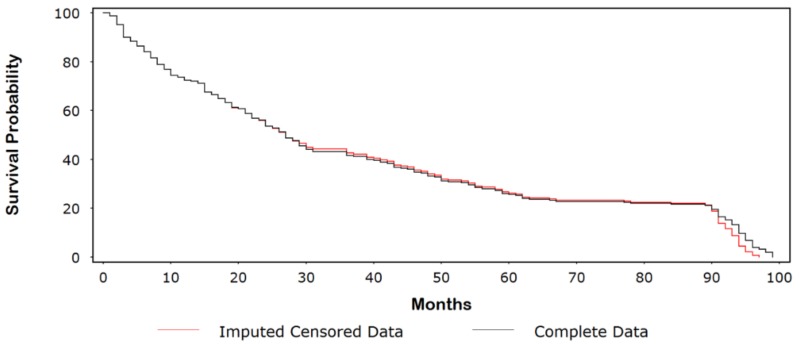
Kaplan-Meir (KM) plot comparing the imputed censored data with the dataset of complete follow-up times.

**Figure 3:**
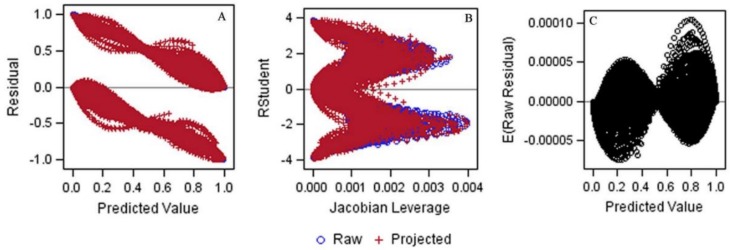
Diagnostic plots. (**A**) Residuals by predicted values; (**B**) Studentized residuals (RStudent) by Jacobian leverages; (**C**) Expected raw residuals by predicted values.

Consider the data in Table A1, which provides the event and censored follow-up times for 250 cancer patients undergoing treatment and their simulated complete values (for illustrative purposes). Approximately 10% of the follow-up times were censored, with the majority of these values occurring early in the study. Figure 1 shows a Kaplan-Meir (KM) plot comparing the original censored data with the dataset of complete follow-up times. The probability of surviving 5 years (60 months) was ~26% for the original censored data compared with ~18% for the complete dataset. In Figure 2, we compare the complete dataset with the imputed censored dataset. At 5 years, rounding to the nearest whole number, we see that the survival times are identical (i.e., 26%). Indeed, the curves are similar until ~90 months, at which point the survival times for the imputed censored time curve are divergently lower than those for the complete dataset. In Figure 3A–C, we observed a few outlying values that indicate a nominal departure from the underlying model goodness-of-fit. 

### 3.2. Generating the Jump-Point Plot

**Figure 4:**
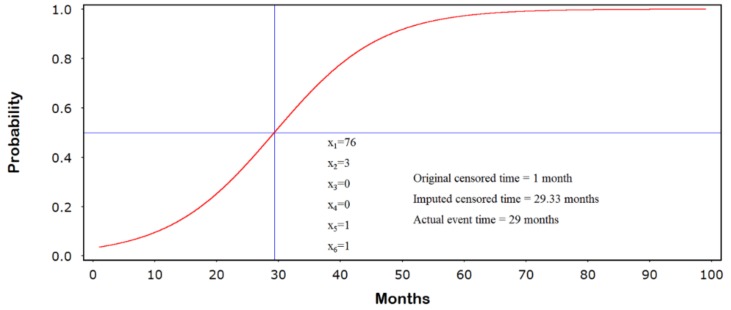
Jump-point plot.

The jump-point plot corresponding to the covariate pattern

(5) $$(x_{1}=76,\;x_{2}=3,x_{3}=0,\;x_{4}=0,\;x_{5}=1,\;x_{6}=0)$$

is shown in Figure 4. Here, the variables $\left(x_{1,}x_{2,}\ldots ,x_{6}\right)$ correspond to age (years), grade (I-IV), lymph node invasion (1 = yes, 0 = no), positive margins (1 = yes, 0 = no), no hormone therapy (1 = yes, 0 = no), and no radiation therapy (1 = yes, 0 = no), respectively. We see that the imputed censored follow-up time of 29.33 months closely matches the actual event time of 29 months. This observation was originally censored at 1 month.

### 3.3. SAS Code

**Figure 5:**
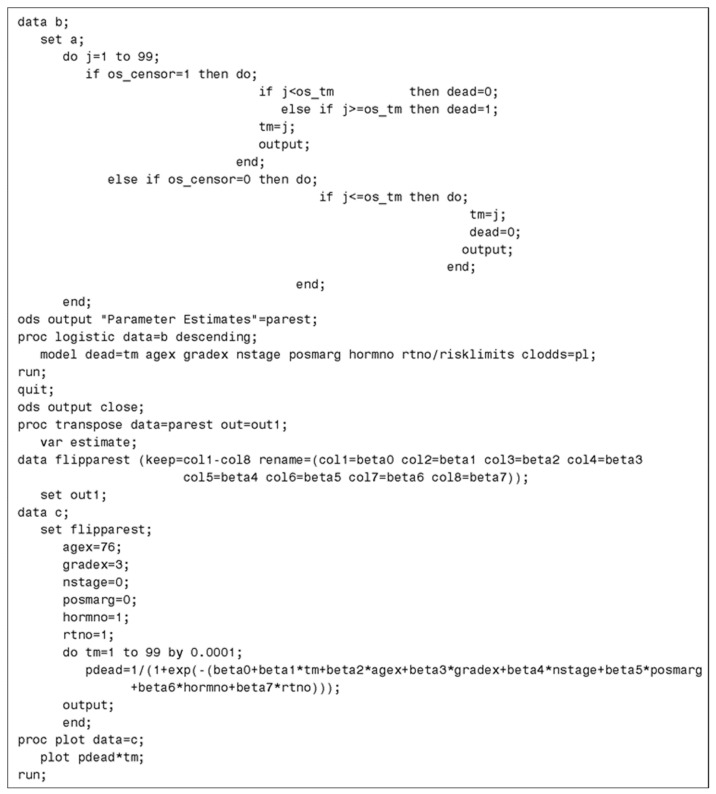
SAS code used to generate jump-point plot.

The SAS code used to generate the jump-point plot is shown in Figure 5. In this code, it is assumed that the data contained in Appendix A has been previously read into the dataset “a”. The counting process variables are created in dataset “b” and then modeled using the “PROC LOGISTIC” procedure. The predicted probabilities generated by this procedure are plotted against time (ranging from 1 to 99 months) to obtain the jump-point plot. Analyses were performed using SAS Version 9.4. (SAS Institute, Cary, NC, USA).

## 4. Discussion

In this manuscript, we have introduced a simple method to partially correct for non-ignorable early censoring. By rearranging the data as a counting process, we are able to account for the follow-up times of all patients, regardless of their censoring status. For example, if a patient is censored at 50 months, the counting process creates 50 observations corresponding to each month and accordingly assigns the value of 0 to an indicator variable. On the other hand, if the patient had an event at 50 months, the counting process would create 49 observations with the indicator variable set to 0 and similarly create new observations for each month thereafter until the last month of the study, but instead assigning the value of 1 to the indicator variable.

These counting responses are then analyzed with logistic regression. In addition to including outcome-related covariates, a variable denoting the time (e.g., month) associated with the indicator variable is added to the model. The predicted value generated by the model for a particular covariate pattern (associated with a censored observation) is then plotted against the time variable (spanning each month of the study) to obtain a jump-point plot. Dropping a line from the midpoint of this plot to the *x*-axis gives an imputed censored follow-up time. We then replace the original censored time with this value if it is the larger of the two values. Additionally, the imputed value is constrained by a natural upper bound to prevent impossible censored follow-up times (e.g., the value must be consistent with a patient’s maximum biologic lifespan).

An important aspect of this technique is identifying a well-fitting model for the counting process data so that it is able to accurately predict if the outcome is 0 or 1. Understanding the dynamics of the disease or process under study will aid in the selection of appropriate outcome-related covariates. However, formally testing for model goodness-of-fit is not practical given the highly correlated nature of the counting process data. In theory, while it may be possible to assess model goodness-of-fit for dependent data using a robust “Huber-White” approach, the regularity conditions for such estimates are quite stringent [15,16,17].

Instead, we recommend using diagnostic plots to rule out ill-fitting models [9,10,11]. As shown in our example, when the Jacobian leverages $\left({\hat{j}}_{i,k}\right)$ (defined as the instantaneous rate of change in the $\left(i^{th}\right)$ predictive value with respect to the $\left(k^{th}\right)$ outcome) were plotted against the studentized residuals, we observed outlying values towards the upper right hand side of this plot (Figure 3B). Correspondingly, we also noticed the presence of extreme points in the lower right-hand side of Figure 3A (which plots predicted values against their residuals) and the upper right portion of Figure 3C (which plots predictive values against their expected raw residuals). Nonetheless, the plots provided in Figure 3A–C were relatively symmetrical with respect to the positive and negative residuals, suggesting that the logistic regression model provided a reasonable fit to the counting process data [18].

The advantage of using a counting process approach is that the imputed censored follow-up times, when appropriately constructed, will better reflect the survival prospects of those who continued in the study. However, because the method is model based, it may not be suitable for small datasets or those lacking a set of reasonably predictive covariates. Additionally, it may not always be possible to identify a well-fitting model if there are abrupt changes in the hazard function of the underlying data. The method at hand should not be used if patients who enroll later in study survive longer (e.g., treatment improves over time) or if enrollment criteria change over the course of the study (e.g., worst patients are excluded midway through recruitment) [3]. 

## 5. Conclusions

Overall, the best means for handling informative censoring is to avoid the problem in the first place. Careful planning at the study design stage, routine patient monitoring, and implementing proactive strategies to minimize patient dropout are some important steps to ensure the fidelity of a survival time study.

While it was beyond the scope of the current manuscript, it will be informative in future analyses to compare our method with other approaches for dealing with censored values, especially highlighting best- and worst-case scenarios [3,19,20,21,22,23,24].

## Appendix A

**Table A1:** Example Cancer Dataset (*N* = 250). A = Observation number, B = Age, C = Grade, D = Lymph node invasion, E = Positive margin, F = No hormonal therapy, G = No radiation therapy, H = Actual follow-up time, I = Original censored time, J = Imputed censored time, K = Censoring variable.

**A**	**B**	**C**	**D**	**E**	**F**	**G**	**H**	**I**	**J**	**K**	**A**	**B**	**C**	**D**	**E**	**F**	**G**	**H**	**I**	**J**	**K**	**A**	**B**	**C**	**D**	**E**	**F**	**G**	**H**	**I**	**J**	**K**
1	93	4	1	1	1	1	1	1	--	1	36	96	4	1	0	1	1	6	6	--	1	71	99	3	0	0	1	1	14	14	--	1
2	90	3	1	1	1	1	1	1	--	1	37	93	3	1	0	1	1	6	6	--	1	72	83	2	1	1	1	1	14	14	--	1
3	89	4	1	1	1	1	1	1	--	1	38	94	3	1	0	0	1	6	6	--	1	73	85	4	1	1	0	1	15	15	--	1
4	89	4	1	0	1	1	2	2	--	1	39	93	3	1	1	1	1	6	6	--	1	74	83	3	1	1	0	1	15	15	--	1
5	86	3	1	1	1	0	2	2	--	1	40	97	3	1	1	1	1	6	6	--	1	75	81	3	1	0	1	1	15	15	--	1
6	86	3	1	0	1	1	2	2	--	1	41	97	3	1	0	1	1	7	7	--	1	76	98	1	1	0	0	1	15	15	--	1
7	95	4	1	1	1	1	2	2	--	1	42	97	2	1	0	1	1	7	7	--	1	77	94	3	1	0	0	1	15	15	--	1
8	90	3	1	1	1	1	2	2	--	1	43	86	3	1	1	1	1	7	7	--	1	78	93	4	1	0	0	0	15	15	--	1
9	86	3	0	1	1	1	2	2	--	1	44	85	3	1	0	1	1	7	7	--	1	79	90	2	1	0	1	1	15	15	--	1
10	84	4	1	0	0	1	2	2	--	1	45	91	3	1	0	1	1	7	7	--	1	80	88	3	1	0	1	1	15	15	--	1
11	82	4	1	0	1	1	2	2	--	1	46	94	3	1	1	1	1	7	7	--	1	81	82	3	1	0	1	1	15	15	--	1
12	91	2	1	1	1	1	2	2	--	1	47	86	3	1	0	1	1	8	8	--	1	82	85	4	1	1	0	0	16	16	--	1
13	96	4	1	0	0	1	3	3	--	1	48	86	4	1	0	1	1	8	8	--	1	83	88	2	1	0	0	0	16	16	--	1
14	95	3	1	0	1	1	3	3	--	1	49	83	1	1	1	0	1	8	8	--	1	84	82	3	1	0	1	0	16	16	--	1
15	89	2	1	1	1	1	3	3	--	1	50	96	3	1	1	1	1	8	8	--	1	85	84	4	1	1	0	0	17	17	--	1
16	88	4	1	0	1	0	3	3	--	1	51	94	4	1	0	0	1	8	8	--	1	86	90	2	1	1	0	1	17	17	--	1
17	91	3	1	1	0	1	3	3	--	1	52	91	3	1	1	1	1	8	8	--	1	87	93	3	1	0	1	0	17	17	--	1
18	91	3	1	1	1	1	3	3	--	1	53	99	3	1	1	1	1	8	8	--	1	88	90	4	1	1	1	1	17	17	--	1
19	90	4	1	1	1	1	3	3	--	1	54	93	4	1	1	0	0	9	9	--	1	89	88	3	0	0	1	0	18	18	--	1
20	98	4	1	0	1	1	3	3	--	1	55	92	2	1	1	0	1	9	9	--	1	90	97	3	1	1	1	1	18	18	--	1
21	94	3	1	1	1	1	3	3	--	1	56	98	2	1	0	1	1	9	9	--	1	91	94	4	1	1	0	0	18	18	--	1
22	96	3	1	0	1	1	3	3	--	1	57	83	4	1	1	0	1	9	9	--	1	92	85	2	1	0	1	1	18	18	--	1
23	88	3	1	0	1	1	3	3	--	1	58	48	3	1	0	1	1	9	9	--	1	93	84	4	1	0	1	1	19	19	--	1
24	95	3	0	1	1	1	3	3	--	1	59	87	2	1	1	1	1	10	10	--	1	94	83	2	1	1	1	1	19	19	--	1
25	83	3	1	0	1	1	3	3	--	1	60	83	4	1	1	0	1	10	10	--	1	95	99	4	1	0	0	1	19	19	--	1
26	88	3	1	0	1	1	4	4	--	1	61	83	3	1	0	0	0	10	10	--	1	96	93	3	1	1	0	1	19	19	--	1
27	95	4	1	1	1	1	4	4	--	1	62	88	4	0	1	1	1	10	10	--	1	97	83	2	1	0	0	0	19	19	--	1
28	95	2	1	1	0	1	4	4	--	1	63	97	2	1	0	1	1	10	10	--	1	98	81	3	0	0	1	1	20	20	--	1
29	92	3	1	1	0	0	4	4	--	1	64	87	3	1	1	1	1	10	10	--	1	99	87	3	1	1	0	1	21	21	--	1
30	96	4	1	0	1	1	5	5	--	1	65	89	4	1	1	1	1	11	11	--	1	100	85	4	1	1	1	1	21	21	--	1
31	96	3	1	0	1	0	5	5	--	1	66	89	2	1	1	1	1	11	11	--	1	101	85	3	1	1	0	1	21	21	--	1
32	90	3	1	1	0	1	5	5	--	1	67	84	2	0	0	0	1	12	12	--	1	102	82	3	1	0	0	1	21	21	--	1
33	89	4	1	1	1	1	5	5	--	1	68	94	4	1	0	0	1	12	12	--	1	103	80	3	1	1	1	1	21	21	--	1
34	87	3	1	0	1	1	5	5	--	1	69	97	3	1	1	0	1	12	12	--	1	104	81	2	1	1	1	1	22	22	--	1
35	97	2	1	1	1	1	6	6	--	1	70	96	2	1	1	0	1	13	13	--	1	105	80	4	1	0	0	1	22	22	--	1
**A**	**B**	**C**	**D**	**E**	**F**	**G**	**H**	**I**	**J**	**K**	**A**	**B**	**C**	**D**	**E**	**F**	**G**	**H**	**I**	**J**	**K**	**A**	**B**	**C**	**D**	**E**	**F**	**G**	**H**	**I**	**J**	**K**
106	91	3	0	1	0	1	22	22	--	1	141	78	2	0	0	0	0	31	31	--	1	176	74	4	1	0	0	1	54	54	--	1
107	75	3	1	0	1	0	22	22	--	1	142	64	4	1	0	0	0	31	31	--	1	177	52	1	0	0	0	1	55	55	--	1
108	72	4	0	0	1	1	22	22	--	1	143	69	3	1	0	1	1	36	36	--	1	178	69	3	1	0	0	0	55	55	--	1
109	83	3	1	1	0	0	23	23	--	1	144	68	2	1	0	0	0	36	36	--	1	179	41	3	0	0	1	1	55	55	--	1
110	79	3	1	1	1	1	23	23	--	1	145	62	2	1	0	0	0	36	36	--	1	180	64	3	1	0	0	1	56	56	--	1
111	79	4	1	0	0	1	24	24	--	1	146	58	3	1	0	1	1	36	36	--	1	181	64	2	1	0	1	1	58	58	--	1
112	78	3	1	1	0	1	24	24	--	1	147	69	4	1	0	0	1	37	37	--	1	182	58	3	1	0	1	1	58	58	--	1
113	75	4	1	0	0	1	24	24	--	1	148	68	3	0	0	0	1	39	39	--	1	183	53	2	0	1	1	1	59	59	--	1
114	84	3	1	0	0	1	24	24	--	1	149	65	3	1	0	0	1	39	39	--	1	184	53	3	1	0	0	1	59	59	--	1
115	78	2	1	1	1	1	24	24	--	1	150	65	3	1	1	0	0	39	39	--	1	185	58	1	0	0	0	0	59	59	--	1
116	78	2	1	0	1	1	24	24	--	1	151	68	3	1	1	1	0	40	40	--	1	186	54	3	1	0	0	1	60	60	--	1
117	79	3	1	0	0	1	25	25	--	1	152	68	4	1	0	1	1	41	41	--	1	187	54	2	0	0	1	1	61	61	--	1
118	73	3	1	1	1	1	25	25	--	1	153	63	3	1	1	0	1	41	41	--	1	188	67	3	1	1	0	0	62	62	--	1
119	73	4	1	0	0	1	26	26	--	1	154	62	3	0	0	1	0	42	42	--	1	189	66	2	1	0	0	1	62	62	--	1
120	72	2	1	0	0	0	26	26	--	1	155	68	1	1	0	1	1	43	43	--	1	190	53	2	0	0	0	0	62	62	--	1
121	75	2	1	0	0	0	26	26	--	1	156	68	2	0	0	0	0	43	43	--	1	191	56	3	1	1	1	1	63	63	--	1
122	69	3	1	1	1	0	26	26	--	1	157	69	2	0	0	1	1	43	43	--	1	192	53	2	1	0	0	1	66	66	--	1
123	89	2	1	0	0	1	27	27	--	1	158	62	3	1	0	1	1	43	43	--	1	193	49	3	1	1	1	1	67	67	--	1
124	78	3	1	0	0	0	27	27	--	1	159	67	3	0	1	1	1	44	44	--	1	194	69	2	1	0	1	1	77	77	--	1
125	78	4	1	1	1	1	27	27	--	1	160	68	2	1	0	1	1	45	45	--	1	195	69	2	0	1	0	0	78	78	--	1
126	72	3	1	1	0	1	27	27	--	1	161	64	3	1	0	0	1	46	46	--	1	196	54	2	1	0	1	1	84	84	--	1
127	63	2	1	0	1	1	27	27	--	1	162	62	2	1	1	0	0	46	46	--	1	197	49	2	1	0	0	1	89	89	--	1
128	62	4	1	1	0	1	27	27	--	1	163	64	3	0	0	0	0	46	46	--	1	198	40	3	0	1	1	1	90	90	--	1
129	79	3	1	0	0	1	28	28	--	1	164	61	3	0	0	1	1	47	47	--	1	199	40	3	1	1	0	0	90	90	--	1
130	73	3	1	0	1	0	28	28	--	1	165	58	4	1	1	0	0	48	48	--	1	200	40	2	1	0	1	1	90	90	--	1
131	73	2	1	0	0	0	28	28	--	1	166	52	3	0	0	0	1	48	48	--	1	201	45	2	1	1	1	1	90	90	--	1
132	76	3	0	0	1	1	29	1	29.33	0	167	51	2	1	0	0	1	48	48	--	1	202	46	3	1	1	1	1	91	2	73.22	0
133	79	4	1	0	0	1	29	4	18.88	0	168	51	2	1	0	1	1	49	49	--	1	203	47	2	0	0	0	1	91	91	--	1
134	84	4	1	0	0	1	29	29	--	1	169	53	3	1	0	0	1	50	50	--	1	204	46	2	1	0	0	1	91	91	--	1
135	82	2	0	0	0	1	29	29	--	1	170	81	3	0	0	0	1	50	50	--	1	205	49	1	1	0	1	1	91	91	--	1
136	78	2	1	0	1	1	29	3	26.73	0	171	77	3	1	1	0	0	50	50	--	1	206	50	1	0	1	0	1	91	91	--	1
137	78	3	0	0	0	1	30	30	--	1	172	44	3	1	0	1	1	50	50	--	1	207	47	2	1	1	1	1	91	91	--	1
138	73	4	0	0	0	1	30	30	--	1	173	68	3	1	0	0	1	51	51	--	1	208	41	2	1	0	1	0	91	91	--	1
139	60	3	1	0	0	0	30	30	--	1	174	72	3	0	1	0	0	53	53	--	1	209	46	2	0	1	0	0	91	91	--	1
140	71	2	1	1	0	0	30	30	--	1	175	87	3	0	1	1	1	54	54	--	1	210	46	2	0	0	1	1	92	92	--	1
**A**	**B**	**C**	**D**	**E**	**F**	**G**	**H**	**I**	**J**	**K**	**A**	**B**	**C**	**D**	**E**	**F**	**G**	**H**	**I**	**J**	**K**											
211	49	3	1	0	1	1	92	92	--	1	246	47	1	0	0	1	1	99	3	85.42	0											
212	52	2	1	0	1	1	92	92	--	1	247	43	2	0	0	1	1	99	10	86.12	0											
213	47	2	0	0	1	1	93	4	79.90	0	248	42	1	1	0	1	1	99	5	88.18	0											
214	48	2	1	0	0	0	93	93	--	1	249	42	1	1	0	0	0	99	2	90.24	0											
215	44	2	1	1	0	1	93	93	--	1	250	46	1	0	0	0	1	99	4	91.71	0											
216	50	3	1	0	0	1	93	93	--	1																						
217	40	1	0	0	0	1	93	93	--	1																						
218	40	3	1	0	1	1	94	2	80.26	0																						
219	56	2	1	1	0	1	94	94	--	1																						
220	51	2	1	0	1	1	94	94	--	1																						
221	48	2	0	0	0	1	94	94	--	1																						
222	51	1	0	0	1	1	94	94	--	1																						
223	51	2	1	1	1	1	94	94	--	1																						
224	52	1	1	1	1	1	94	94	--	1																						
225	48	2	0	1	0	1	94	6	85.36	0																						
226	42	2	1	0	0	1	94	1	87.41	0																						
227	49	1	0	0	1	1	95	95	--	1																						
228	49	2	1	1	1	1	95	95	--	1																						
229	51	2	1	0	1	1	95	2	68.68	0																						
230	51	3	0	0	1	1	95	95	--	1																						
231	45	1	1	0	0	0	95	8	85.58	0																						
232	45	1	1	1	0	1	95	4	90.53	0																						
233	43	2	0	0	0	0	95	2	88.18	0																						
234	47	1	1	1	0	1	96	3	87.43	0																						
235	42	1	1	0	1	1	96	96	--	1																						
236	46	3	0	0	1	1	96	1	75.95	0																						
237	47	2	1	1	1	1	96	96	--	1																						
238	45	2	0	0	1	1	96	4	83.01	0																						
239	46	1	1	1	1	1	96	7	84.24	0																						
240	46	2	0	1	0	1	96	1	88.47	0																						
241	46	1	1	0	0	1	97	7	86.70	0																						
242	46	2	1	1	1	1	97	97	--	1																						
243	46	3	0	1	1	1	98	4	78.22	0																						
244	46	2	0	0	1	1	98	5	81.46	0																						
245	46	3	1	0	0	1	98	2	75.68	0																						
